# Pulsed Current Electrodeposition of Ag Nanoparticles on Bamboo-like TiO_2_ Nanotubes for Surface-Enhanced Raman Scattering Substrates

**DOI:** 10.1021/acsomega.5c10021

**Published:** 2025-12-11

**Authors:** Marcos Luna Cervantes, Ismael Garcia-Ramírez, Erick Octavio Santos Santiago, Diana Jiménez Girón, José Luis Zamora Navarro, Antonio García Chavez, Leandro García-González, Adriana Báez-Rodríguez, Julián Hernández Torres, Luis Zamora-Peredo

**Affiliations:** 1 Centro de Investigación en Micro y Nanotecnología, 27870 Universidad Veracruzana, Av. Adolfo Ruiz Cortines 455, col. Costa Verde, Boca del Río 94294, México; 2 Doctorado en Ciencias e Ingeniería, 27758 Universidad Autónoma de Baja California, Carretera Transpeninsular Ensenada - Tijuana 3917, col. Playitas, Ensenada 22860, México

## Abstract

In this work, we investigate the performance of surface-enhanced Raman scattering (SERS) substrates based on bamboo-like TiO_2_ nanotubes (BTNTs) functionalized with silver nanoparticles (AgNPs) through pulsed-current electrodeposition (50 ms ON/250 ms OFF). The BTNT arrays, fabricated via alternating-voltage anodization, exhibit segmented morphologies that enhance nanoparticle anchoring and promote the dense generation of electromagnetic hotspots. Systematic optimization of deposition parameters revealed that 400 deposition cycles at a current density of 5 mA/cm^2^ yielded the most favorable combination of nanoparticle distribution, structural morphology, and SERS activity. Under these conditions, the substrates achieved an analytical enhancement factor (AEF) of 1 × 10^6^ and enabled the experimental detection of methylene blue down to 1 × 10^–8^ M (theoretical LoD 1 × 10^–9^ M). This study demonstrates a cost-effective, scalable, and tunable fabrication strategy for highly active SERS platforms, offering significant potential for practical sensing applications.

## Graphical abstract

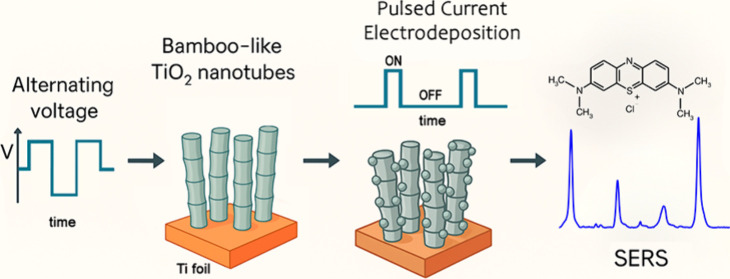

## 1 Introduction

Surface-enhanced Raman scattering (SERS) has emerged as a highly sensitive analytical technique for detecting chemical and biological analytes.^1–4^ Its enhancement capability, primarily driven by localized surface plasmon resonance (LSPR) in metallic nanostructures, can amplify Raman signals by several orders of magnitude, enabling single-molecule detection under optimized conditions.^5,6^ Titanium dioxide nanotubes (TNT) substrates are frequently functionalized with silver nanostructures (AgNSs) for use in SERS applications. The morphological characteristics of the substrates significantly influence how AgNSs adhere and, consequently, modify the SERS response.

Anodization is one of the most widely used methods to fabricate TNT, due to its simplicity, cost-effectiveness, reproducibility, morphological tunability, and scalability.^7^ Numerous studies have been published regarding this electrochemical technique, including the parameters involved and their impact on the resulting morphology.^8^ In terms of technical implementation, anodization can be performed under constant voltage (CV),^9^ alternating voltage (AV),^10^ or constant current (CC)^11^ regimes. To date, few studies have used the AV methodology for SERS applications. Notably, bamboo-like TiO_2_ nanotubes (BTNTs), produced through AV cycling between high and low voltages, exhibit periodic constrictions along their length that enhance nanoparticle immobilization.^12,13^ Despite these advantages, BTNTs have been primarily applied in energy-related fields such as lithium-ion batteries,^14^ dye-sensitized solar cells,^15^ photocatalysis,^16^ hydrogen generation,^17^ and energy storage.^18^

Silver nanostructures can be fabricated into diverse morphologies, including nanoparticles, nanorods, nanosheets, pyramids, flower-like particles, and dendrites.^3,4,19^ Among them, silver nanoparticles (AgNPs) are widely utilized in multifunctional sensing platforms due to their unique physicochemical properties.^20,21^ Their optical response, governed by LSPR, is highly dependent on particle geometry and the dielectric environment. Consequently, AgNPs are ideal candidates for a range of detection technologies, including colorimetric, fluorometric, and SERS-based systems.^22^ Controlling the size, distribution, and density of AgNPs remains critical for achieving high SERS enhancement.^23–25^ Various deposition strategies have been employed to functionalize TiO_2_ nanotubes with AgNPs, including chemical methods in aqueous media, constant-current electrodeposition,^26^ photodeposition,^27^ and DC magnetron sputtering.^28^ However, these methodologies have limitations, as they require specialized equipment, involve the use of hazardous materials, and necessitate the use of seed particles, templates, or multiple stabilizing agents, which increase production costs.^19^ For instance, constant-current electrodeposition often results in uncontrolled nucleation and nanoparticle agglomeration due to sustained ion depletion.^29^ Photodeposition typically yields low particle densities and poor spatial uniformity.^30^ Meanwhile, DC magnetron sputtering requires vacuum systems and often leads to excessive particle clustering.^31^

In contrast, pulsed-current electrodeposition (PCE) offers a versatile and controllable strategy for tailoring nanoparticle nucleation and growth under ambient conditions. The use of intermittent current pulses periodically reconstructs the electrical double layer, mitigating ion depletion and preventing uncontrolled agglomeration.^32–34^ At moderate-to-high silver nitrate concentrations (≥10 mM), short ON pulses (<100 ms) deliver charge with high temporal resolution while suppressing excessive growth, favoring nucleation-dominated processes.^35,36^ The OFF periods further facilitate ionic redistribution and replenishment near the electrode surface, preventing local depletion zones and enabling uniform nucleation across the nanotube array. This dynamic ON–OFF modulation minimizes continuous growth of a few dominant nuclei, thereby reducing the formation of dendritic structures while allowing new nuclei to stabilize and grow in a controlled manner. As a result, PCE enables dense nanoparticle coverage with desirable morphological diversity.

Furthermore, the applied current density has a strong influence on nanoparticle growth dynamics.^37–39^ An optimized current density balances nucleation and growth dynamics, producing well-controlled nanostructures with enhanced SERS activity. A distribution of particle sizes combined with anisotropic morphologies significantly strengthens near-field electromagnetic fields via multipolar plasmon modes mediated by strong interparticle coupling. Such architecture-induced disorder has been shown to substantially amplify Raman signals, even in substrates with high polydispersity.^40^

Despite these advances, to the best of our knowledge, this is the first report on bamboo-like TiO_2_ nanotubes decorated with silver nanoparticles via pulsed-current electrodeposition for SERS applications. While BTNTs have been widely investigated in other fields, their potential as SERS substrates has remained unexplored. Additionally, previous strategies for AgNP functionalization of TiO_2_ nanotubes have primarily focused on smooth-walled architectures, without addressing the advantages of segmented morphologies. In this work, we propose a novel approach that combines BTNTs with pulsed-current deposition, providing a rational pathway for the reproducible and scalable fabrication of SERS substrates.

## 2 Materials and Methods

### 2.1 Preparation of Bamboo-like TiO_2_ Nanotubes

Bamboo-like TiO_2_ nanotubes (BTNTs) were synthesized using a titanium foil (grade II, 5 × 15 × 0.1 mm) as anode, positioned 1 cm apart from a graphite rod cathode (6 mm diameter, StonyLab, USA). Prior to anodization, the titanium foils were cleaned in three sequential 10 min ultrasonic baths of acetone (C_3_H_6_O, Karal, Mex), ethanol (C_2_H_6_O, HYCEL, Mex), and deionized water. The electrodes were partially immersed in an organic electrolyte composed of 0.33 wt % ammonium fluoride (NH_4_F, Meyer, USA), 2% deionized water, and monoethylene glycol (MEG, C_2_H_6_O_2_, Karal, Mex) under magnetic stirring. Anodization was carried out at room temperature (21–25 °C) using an alternating voltage (AV) sequence of 60 and 20 V, applied every 2 min for a total of 30 cycles. The current response was monitored in situ using a UT117C Uni-T multimeter. For comparison, smooth-walled nanotubes were prepared under constant voltage anodization at 60 V for 1 h, corresponding to the effective high-voltage duration of the AV protocol. Following anodization, the samples were rinsed with deionized water and ethanol, and annealed in air at 450 °C for 4 h.

### 2.2 Pulsed Current Electrodeposition of AgNPs

A two-electrode setup was used for PCE of AgNPs, employing the previously anodized substrates as the cathode and a Pt sheet (15 × 15 mm × 0.1 mm, StonyLab, USA) as the anode. PCE was carried out in an aqueous solution containing 10 mM silver nitrate (AgNO_3_, Sigma-Aldrich, USA) and 100 mM sodium nitrate (NaNO_3_, Meyer, USA) at room temperature, alternating 50 ms ON/250 ms OFF time. A custom-built Arduino-based system controlled the pulsed current deposition. The AgNPs were deposited at current densities of 5 and 10 mA/cm^2^, while varying the number of cycles from 100 to 500. After deposition, all substrates were thoroughly rinsed with deionized water and dried in air. Three independent replicates were prepared for each deposition condition to ensure reproducibility of the results.

### 2.3 Methylene Blue Solutions

A standard Methylene Blue dye (MB, C_16_H_18_ClN_3_S, HYCEL, Mex) was employed as the probe molecule to evaluate SERS performance. Three solutions with a concentration of 1 × 10^–4^ to 1 × 10^–8^ M were prepared using ethanol as solvent. For all SERS measurements, a 20 μL aliquot of solution was deposited onto the substrates and dried under ambient conditions.

### 2.4 Raman Spectroscopy, Morphological, and Elemental Characterization

Crystal phase identification and SERS measurements were performed using an Ocean Insights QE Pro Raman spectrometer equipped with a 785 nm excitation laser. The spot size of the focused laser was approximately 500 μm. Surface morphology and elemental composition were examined by field-emission scanning electron microscopy (FESEM, EDS, JEOL JSM-7600F). SEM images were acquired using secondary electrons at 15 kV and a working distance of 5.0 mm. Tube and AgNPs diameters were determined using the ImageJ software (v1.53k), and average values were calculated from at least 30 measurements per sample. Porosity quantification was also performed using the same software.

## 3 Results and Discussion

The time evolution of current density (*J*) during anodization provides valuable insight into the mechanisms governing nanotube formation and morphological transitions. Figure 1a compares the *J*–*t* profiles obtained under two anodization regimes: a constant voltage (CV) mode at 60 V and an alternating voltage (AV) mode cycling between 60 and 20 V, each held for 2 min over 30 cycles. Under CV conditions, the current exhibited a classic exponential decay,^9^ corresponding to the initial growth of a compact oxide layer and subsequent steady-state pore formation. This behavior is consistent with previous reports on self-organized TiO_2_ nanotube arrays, where current decay reflects the progressive thickening of the barrier layer and stabilization of ion transport across the oxide–electrolyte interface. In contrast, the AV regime produced a distinctive profile characterized by periodic current spikes at each 20–60 V transition.^41^ These transients originate from enhanced electric fields that accelerate Ti^4+^ and O^2–^ ion migration, promoting field-assisted oxide dissolution.^42^ During the low-voltage phase, partial reconstruction of the barrier layer reduces ion mobility and temporarily halts tube elongation. Once the high voltage is reapplied, oxide growth resumes at the base of the preformed structures, progressively forming discrete segments. This cyclic growth–interruption mechanism drives the characteristic bamboo-like morphology. In our system, the first 60 V pulse produced a peak current density of 8 mA/cm^2^, which gradually decreased over subsequent cycles due to local fluoride depletion and increasing oxide thickness, both of which limit ion transport. The resulting sawtooth-shaped *J*–*t* curve is consistent with the segmental growth mechanism described above.

**Figure 1:**
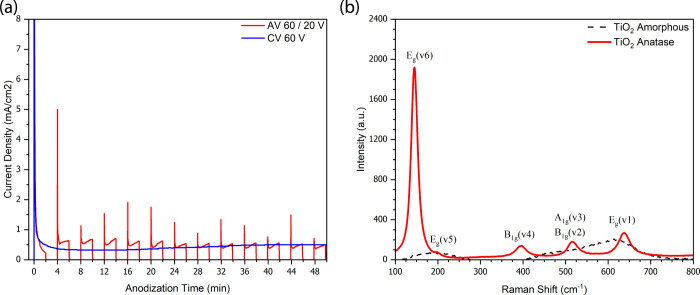
(a) Current density versus time (*J*–*t*) profiles during Ti anodization under constant voltage (blue line, CV) and alternating voltage (red line, AV) and (b) Raman spectra of TiO_2_ nanotubes before and after annealing at 450 °C for 4 h.

Figure 1b shows the Raman spectra recorded before annealing and after annealing at 450 °C, evidencing the crystallization of the TiO_2_ nanotube array. The characteristic TiO_2_ modes E_g_(ν6), E_g_(ν5), B_1g_ (ν4), A_1g_ (ν3) + B_1g_ (ν2), and E_g_ (ν1) were observed at 144, 196, 394, 515, and 636 cm^–1^,^43^ respectively, only after annealing, confirming that the thermal treatment at 450 °C is required to develop the anatase crystalline phase. The absence of peaks at ∼447 and ∼612 cm^–1^ confirms that no rutile domains are present. The mean Raman intensity of the 144 cm^–1^ mode was 2024 ± 145 au, across 16 measurements, with a relative standard deviation (RSD) of 7%, demonstrating good homogeneity. Measurements across three independent substrates yielded 1921 ± 180 au with an RSD of 9% which is good value for reproducibility.

Figure 2a,b shows top-view and close-up SEM images of the bamboo-like TiO_2_ nanotube arrays after anodization and annealing. The micrographs reveal vertically aligned nanotubes exhibiting periodic radial constrictions along their length, providing clear evidence of the morphological modulation induced by the alternating voltage protocol. Image analysis yielded an average inner diameter of 93 ± 7 nm and an outer diameter of 113 ± 14 nm, with an estimated porosity of 39%. The wall thickness was approximately 10 nm, and constriction spacing was relatively uniform across the sample. This segmentation arises from sequential cycles of compact oxide formation and tube elongation, with the frequency and regularity directly linked to the applied voltage sequence.

**Figure 2:**
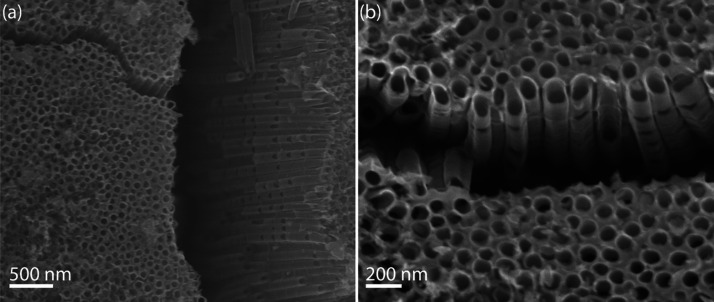
SEM images of bamboo-like TiO_2_ nanotubes formed under AV anodization: (a) top view and (b) tilted close-up view. The nanotubes exhibit periodic radial constrictions along with their length, characteristic of segmental growth driven by voltage cycling.

AgNPs were electrodeposited onto the BTNTs substrates at current densities of 5 and 10 mA/cm^2^. The number of deposition cycles was varied from 100 to 500. The main vibrational modes of Methylene Blue were used for SERS quantification. Four characteristic Raman peaks were consistently observed at 870, 1144, 1412, and 1618 cm^–1^,^1^ corresponding to C–H in-plane bending, C–N asymmetric stretching, and aromatic C=C stretching vibrations, respectively.^4^ Among these, the 1618 cm^–1^ mode, enhanced by its delocalized π-electron system,^44^ showed high reproducibility and signal intensity, making it the most suitable for analytical enhancement factor (AEF) calculations.

Figure 3a shows the SERS spectra obtained at 5 mA/cm^2^ under different deposition cycles. Raman intensity increased progressively with cycle number, reaching a maximum at 400 cycles. This behavior indicates that controlled nanoparticle growth and uniform distribution at this current density favor the formation of abundant electromagnetic hotspots, leading to strong SERS enhancement.^45,46^

**Figure 3:**
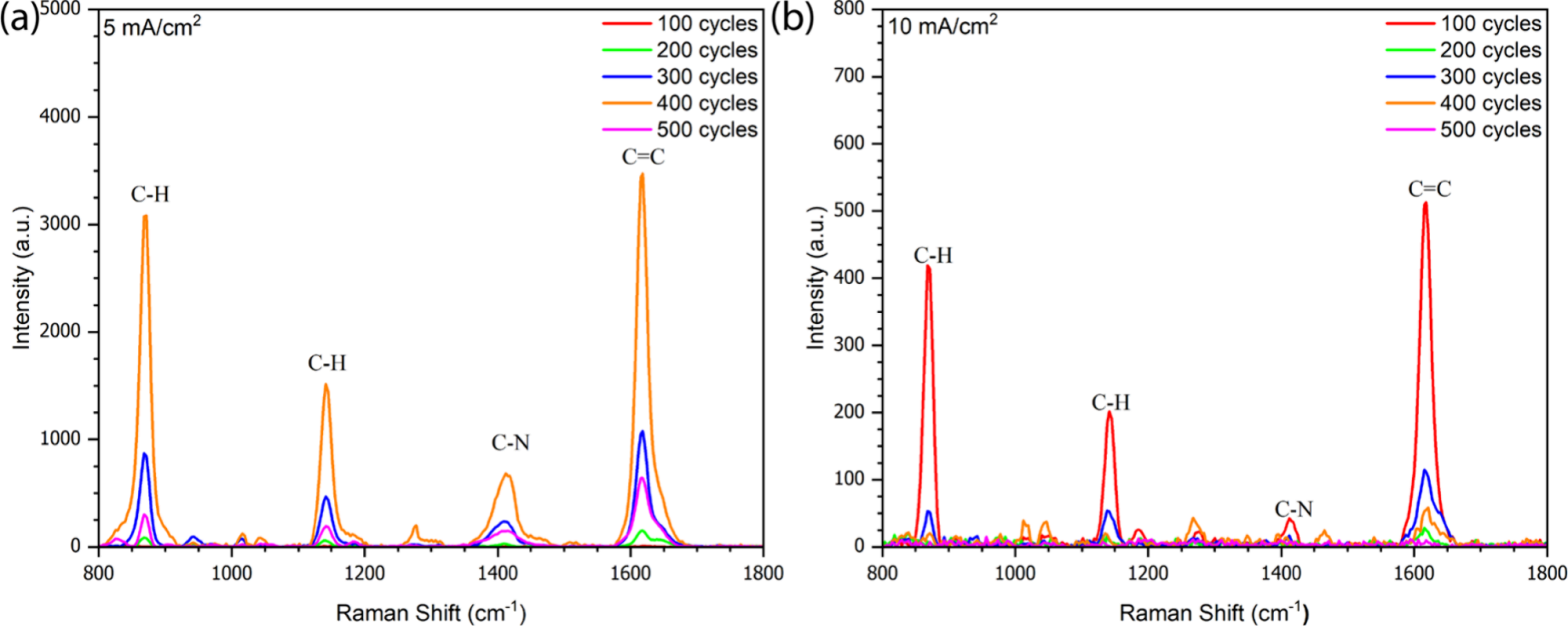
SERS spectra of MB (10^–8^ M) on BTNT substrates after AgNP electrodeposition at (a) 5 mA/cm^2^ and (b) 10 mA/cm^2^ with varying cycle numbers.

In contrast, Figure 3b shows that at 10 mA/cm^2^, SERS intensity increased only slightly at 100 cycles and then declined as the cycle number increased. This behavior suggests that the higher current density accelerates nucleation and growth, causing uncontrolled AgNPs agglomeration. The resulting overgrowth reduces interparticle gaps—essential for localized surface plasmon resonance (LSPR)—and consequently decreases hotspot density. Similar phenomena associated with excessive metal loading have been reported in previous SERS studies.^47,48^ Importantly, in pulsed electrodeposition, very low pulse numbers (<100 cycles) fall into a regime of extremely low delivered charge, where stochastic single-nucleus events dominate and reproducibility collapses. This behavior has been widely documented in pulse-current plating and nanoscale electrodeposition studies, where a minimum number of pulses is needed before the system enters a statistically meaningful growth domain.^33,35,49,50^ For this reason, the 10 mA cm^–2^ branch was intentionally started at 100 cycles as a fixed comparative reference point, and not as an optimization domain.

Additionally, a brownish precipitate was observed in the electrolyte after deposition, suggesting undesired side reactions or uncontrolled silver reduction in solution. These signs of instability, along with the decreasing SERS signal, prompted a reassessment of the current density. The 5 mA/cm^2^ was therefore selected for the next steps.

To correlate SERS performance with morphological features, SEM analysis was conducted on representative samples. After 100 deposition cycles (Figure 4a), AgNPs were sparsely distributed across the BTNT surface, with wide interparticle gaps that limited hotspot formation and resulted in weak Raman signals. High-magnification images (Figure 4b) showed nanoparticles with an average diameter of 57 ± 15 nm and an interparticle spacing of 239 ± 113 nm. In contrast, substrates prepared with 400 cycles (Figure 4c) exhibited dense AgNP decoration over the mouths and walls of the nanotubes. Higher-magnification analysis revealed a polydisperse nanoparticle population with occasional irregular geometries and an average diameter of 113 ± 69 nm. Although not monodisperse, the particles were sufficiently close to enable strong plasmonic coupling and the formation of numerous SERS-active hotspots.

**Figure 4:**
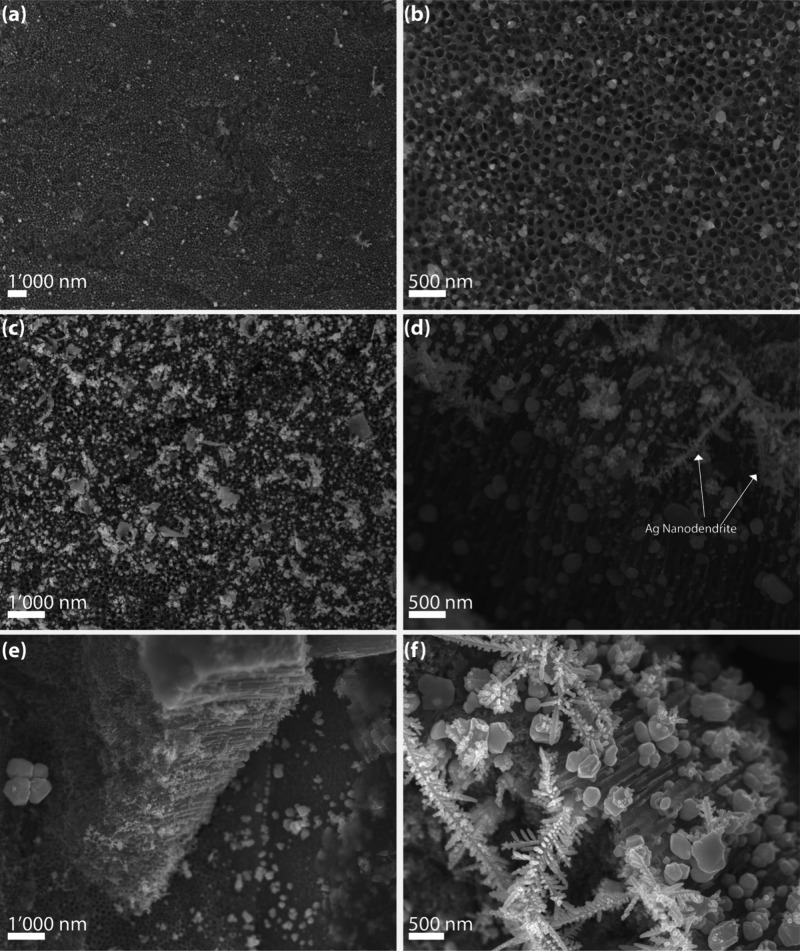
(a, b) SEM images of BTNT substrates after 100 deposition cycles at 5 mA/cm^2^, showing low AgNP coverage and wide interparticle spacing; (c, d) substrates after 400 cycles exhibit dense, polydisperse nanoparticle distribution with occasional dendritic growth; (e, f) 10 mA/cm^2^, 100 and 200 cycles, respectively, massive Ag coalescence and onset of dendritic/branch-like domains, consistent with the collapse of SERS efficiency at this current density.

Cross-sectional SEM images (Figure 4d) confirmed that nanoparticle growth was not restricted to the surface but extended along the inner walls of the nanotubes. This vertical distribution results from the OFF period of the pulsed deposition, which facilitates Ag^+^ diffusion into the tubular channels.^51^ Occasional branched or dendritic features were also observed, likely originating from local electric field gradients and ion depletion within the confined geometry.^52^ Their limited presence indicates that the selected pulse conditions (50 ms ON/250 ms OFF, 5 mA/cm^2^) effectively control nucleation and growth. Moreover, these anisotropic structures may enhance SERS efficiency by supporting multipolar plasmonic interactions.^53^ The full morphological evolution for the 5 mA/cm^2^ branch (100–500 cycles) is provided in Supplementary Figure S1. Additionally, Figure 4e–f shows the morphology obtained at 10 mA/cm^2^ after 100 and 200 cycles respectively, where the onset of particle agglomeration is already evident, in agreement with the poor SERS performance observed at this current density. The size of Ag nanoparticles is governed by the interplay between current density and the total number of deposition cycles. The current density dictates the instantaneous growth rate during each ON pulse, whereas the number of cycles determines the total delivered charge. Therefore, size control in our system arises from these two parameters, while the ON/OFF pulse durations (50 ms/250 ms) are kept constant to maintain the same mass-transport regime.

The segmented geometry of BTNTs further contributes to nanoparticle distribution. The periodic constrictions formed during anodization act as preferential nucleation sites, concentrating the local electric field and enhancing ionic penetration during OFF periods.^10,41^ As a result, AgNPs form not only at the tube openings but also along the inner walls, increasing the density of accessible hotspots. Additionally, the coexistence of spherical, elongated, and irregular nanoparticles at 400 cycles creates a heterogeneous plasmonic environment.^54^ This morphological diversity supports multipolar plasmon resonances and interparticle coupling across multiple length scales, significantly amplifying the local electromagnetic field.^40,55,56^ The combined effects of segmented nanotube architecture and controlled polydispersity explain the superior SERS performance observed under optimized conditions. The correlation between nanoparticle size and Raman intensity as a function of deposition cycle number and current density is summarized in Table 1.

**Table 1:** Average AgNP Diameters and Corresponding SERS Intensity of the 1618 cm^–1^ Raman Mode as a Function of the Number of Deposition Cycles at 5 and 10 mA/cm^2^

	AgNP diameter (nm)		1618 cm^–1^ SERS intensity (a.u.)	
cycles/current density	5 mA/cm^2^	10 mA/cm^2^	5 mA/cm^2^	10 mA/cm^2^
100	57 ± 15	181 ± 40	7 ± 4	513 ± 23
200	59 ± 22	289 ± 75	151 ± 56	115 ± 31
300	79 ± 49	NA	1077 ± 127	58 ± 16
400	113 ± 69	NA	3727 ± 309	28 ± 8
500	168 ± 46	NA	641 ± 345	10 ± 2

The electrochemical processes underlying pulsed-current deposition explain the observed nanoparticle distribution.^29,32,36,39,52,57^ During the ON-period (50 ms), Ag^+^ ions are reduced to metallic silver at the BTNTs surface, as described in eq 1:

$${\mathrm{Ag}}^{+}+e^{-}\to \mathrm{Ag}$$ 1

At the Pt anode, water oxidation sustains charge balance (eq 2):

$$2H_{2}O\to O_{2}+4H^{+}+4e^{-}$$ 2

The supporting electrolyte contributes only through dissociation, maintaining conductivity (eq 3):

$${\mathrm{NaNO}}_{3}\to {\mathrm{Na}}^{+}+{\mathrm{NO}}_{3}^{-}$$ 3

Under localized alkaline conditions, transient intermediates may form (eqs 4 and 5)­

$${\mathrm{Ag}}^{+}+{\mathrm{OH}}^{-}\to \mathrm{AgOH}$$ 4

$$2\mathrm{AgOH}\to {\mathrm{Ag}}_{2}O+H_{2}O$$ 5

which can be subsequently reduced in the next ON-periods (eq 6)­

$${\mathrm{Ag}}_{2}O+H_{2}O+2e^{-}\to 2\mathrm{Ag}+2{\mathrm{OH}}^{-}$$ 6

Complementary to the electrochemical reactions governing Ag^+^ reduction, the segmented architecture of BTNTs biases Ag growth toward preferential nucleation at tube mouths and constrictions during pulsed electrodeposition. Local field crowding in these regions concentrates the current density, and once initial Ag nuclei form, subsequent pulses reinforce deposition at these high-field sites rather than producing isotropic growth. Under pulsed deposition, ON periods promote rapid Ag^+^ reduction, while OFF periods allow partial recovery of the diffusion layer, preventing surface passivation and sustaining nucleation within the tubular channels. This ON/OFF modulation results in polydisperse nanoparticles with occasional anisotropic geometries, consistent with SEM observations at 400 cycles shown in Figure 4c–d. Importantly, the coexistence of spherical, elongated, and irregular AgNPs generates a heterogeneous plasmonic landscape that enhances hotspot density through multipolar plasmon resonances.^49,58–61^

After identifying 400 cycles at 5 mA/cm^2^ as the condition that produced the strongest SERS response, a current density sweep was performed while maintaining the cycle number constant. As shown in Figure 5a, the Raman signal intensity increased progressively with J, reaching a maximum at 5 mA/cm^2^, beyond which the signal intensity began to decrease. This decrease at higher current densities is attributed to excessive nucleation and overgrowth, leading to nanoparticle clustering. SEM analysis at 7 mA/cm^2^ (Figure 5b) confirmed pronounced agglomeration of AgNPs, which compromises plasmonic coupling and reduces hotspot density. These results confirm that 400 cycles at 5 mA/cm^2^ represent the optimal electrodeposition conditions, providing the best balance between nanoparticle distribution, morphology, and SERS performance. Complementary SEM images (Figure S2) show that increasing current density from 3 to 7 mA/cm^2^ at 400 cycles enlarges the Ag nanoparticles, narrows interparticle spacing, and leads to local coalescence—particularly at 7 mA/cm^2^—explaining the concomitant decrease in SERS intensity.

**Figure 5:**
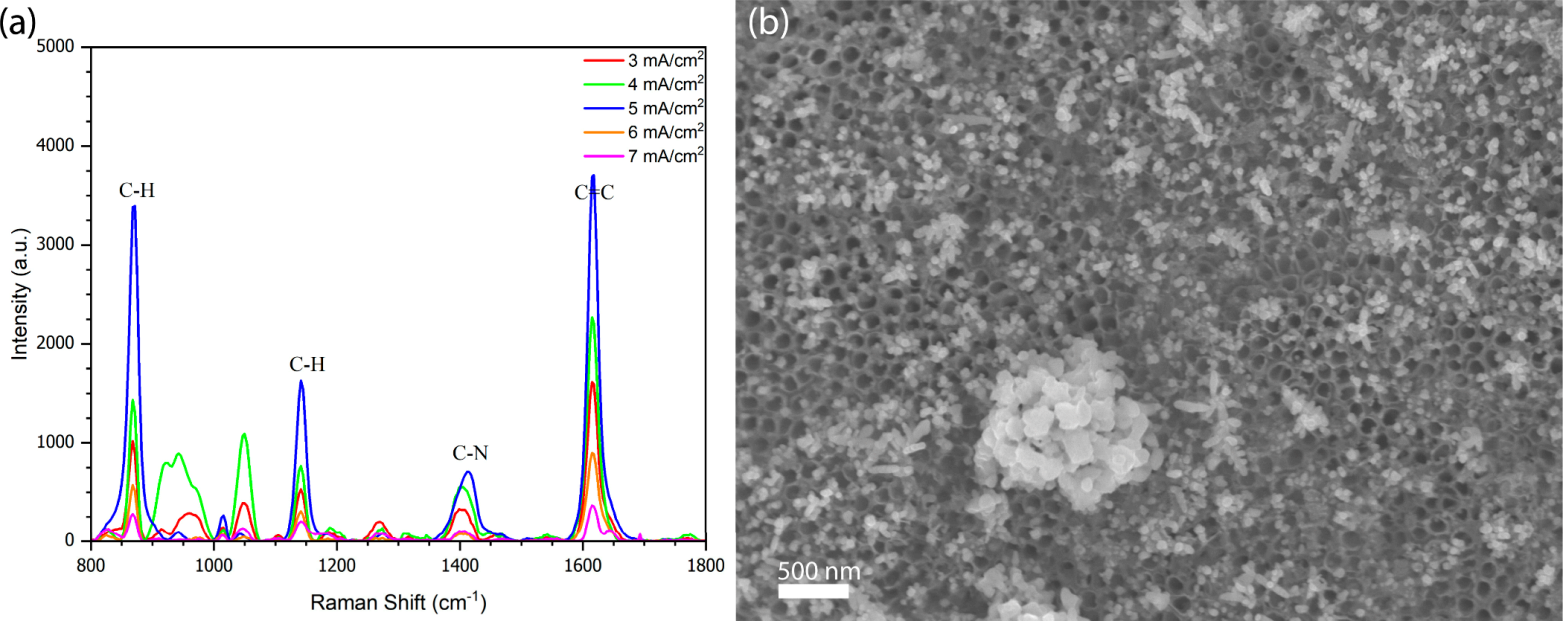
(a) Raman spectra recorded on BTNTs/AgNP substrates after 400 deposition cycles at varying current densities (3–7 mA/cm^2^). (b) SEM image of AgNPs deposited at 7 mA/cm^2^, showing nanoparticle agglomeration that limits plasmonic efficiency.

Figure 6 shows two representative EDS point spectra acquired from the sample electrodeposited at 5 mA/cm^2^ for 400 cycles. In both regions (Spectrum 1 and Spectrum 2), pronounced Ag peaks (∼3.0–3.2 keV) were observed, together with Ti (∼4.5–4.9 keV) and O (∼0.53 keV) signals originating from the underlying TiO_2_. These results confirm the presence and spatial localization of Ag on the anodized TiO_2_ scaffold.

**Figure 6:**
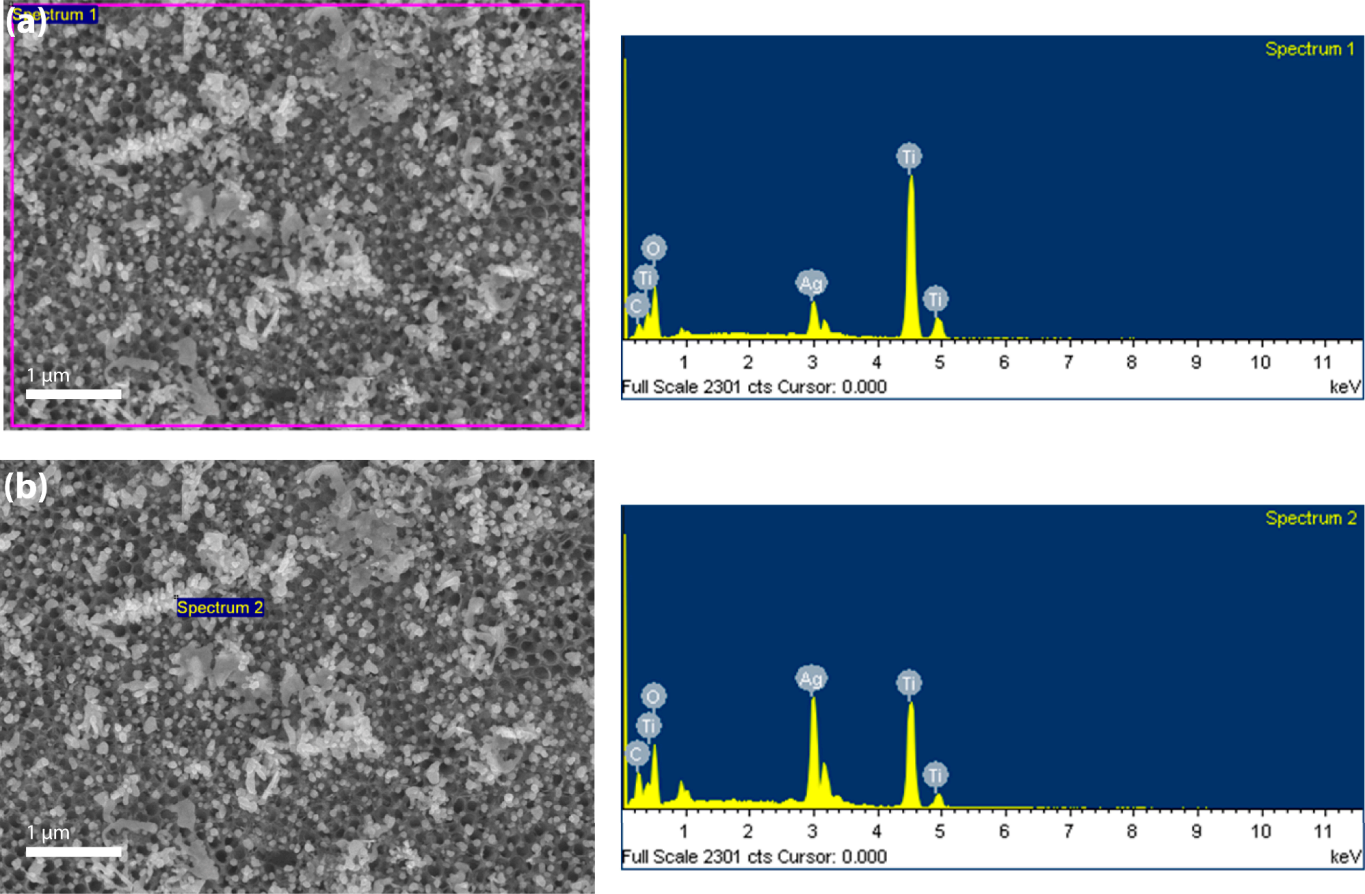
SEM–EDS of the sample electrodeposited at 5 mA/cm^2^ and 400 cycles. (a) SEM top view with the Spectrum 1 acquisition area (left) and the corresponding EDS spectrum (right). (b) SEM top view with Spectrum 2 acquisition point (left) and the corresponding EDS spectrum (right).

To directly evaluate the impact of the anodization protocol on SERS performance, Figure 7 compares the Raman spectra of methylene blue obtained from substrates prepared by CV anodization at 60 V [red curve, Smooth-walled] and AV anodization at 60/20 V [blue curve, Bamboo-like]. The BTNT-based substrate exhibited significantly stronger Raman signals, particularly at the characteristic modes of 870, 1144, 1412, and 1618 cm^–1^, compared with the smooth-walled nanotubes formed under the CV regime. This enhanced response is attributed to the segmented morphology of BTNTs, which provides preferential anchoring sites for AgNPs and promotes a higher density of electromagnetic hotspots. These results confirm that the bamboo-like architecture not only modifies the electrochemical growth dynamics (as shown in Figure 1a) but also leads to significantly enhanced SERS performance relative to conventional TiO_2_ nanotubes. Note that mode-specific intensity redistributions are expected in SERS due to adsorption geometry, charge-transfer contributions, and excitation-wavelength effects; thus, an individual band (e.g., C–N) can appear relatively stronger without indicating a higher overall enhancement. This behavior is consistent with prior SERS reports, including studies on methylene blue.^62,63^ To better visualize the structural differences between both architectures, cross-sectional SEM images are provided in Supplementary Figure S3, showing the uniform walls of the smooth-walled nanotubes (continuous 60 V anodization) and the periodically constricted channels characteristic of the bamboo-like nanotubes (alternating-voltage anodization). In addition, Supplementary Figure S4 presents top-view images of both morphologies before and after Ag electrodeposition, highlighting the presence of TiO_2_ residues on the surface of the smooth-walled nanotubes due to continuous oxide dissolution, whereas the alternating-voltage regime yields a cleaner and more defined surface.

**Figure 7:**
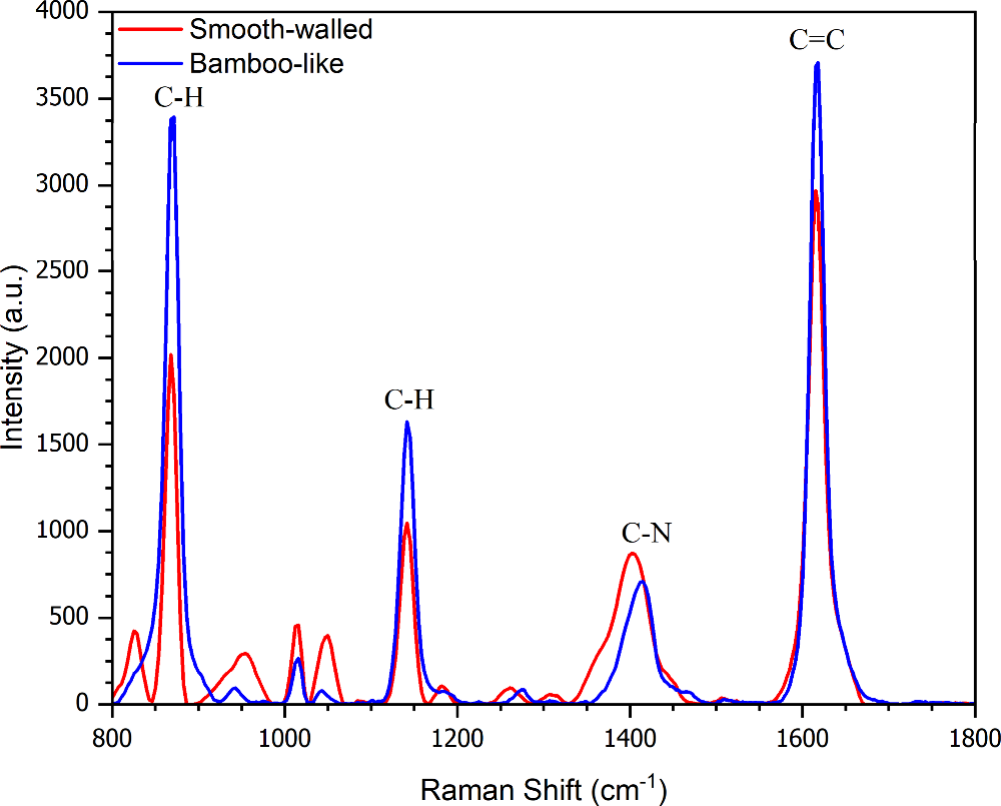
Comparative SERS spectra of methylene blue (1 × 10^–8^ M) obtained on smooth-walled TiO_2_ nanotubes (red curve, constant voltage anodization at 60 V) and on bamboo-like nanotubes (blue curve, alternating voltage at 60/20 V).

Following the optimization of deposition parameters, the condition of 400 cycles at a current density of 5 mA/cm^2^ was selected as optimal for AgNP growth on BTNTs arrays. This configuration offered the best balance between nanoparticle density, morphology, and Raman signal enhancement. The analytical enhancement factor (AEF) was determined using eq 7,^64^ which relates the SERS signal intensity (*I*_SERS_) and analyte concentration (*C*_SERS_) on the active substrate to the corresponding values measured on a nonenhancing reference (*I*_ref_ and *C*_ref_):

$$\mathrm{AEF}=\left(\frac{C_{\mathrm{ref}}}{C_{\mathrm{SERS}}}\right)\left(\frac{I_{\mathrm{SERS}}}{I_{\mathrm{ref}}}\right)$$ 7

For AEF calculation, the reference substrate corresponded to the TiO_2_ nanotube array without Ag deposition, measured under identical optical conditions (same laser power, exposure time, and analyte concentration). No detectable Raman signal of methylene blue was observed on the bare TiO_2_ surface, confirming that the enhancement originates exclusively from the plasmonic Ag nanostructures.

AEF values were calculated for four characteristic Raman modes of MB: 870, 1144, 1412, and 1618 cm^–1^. Among them, the band at 1618 cm^–1^ was selected as a benchmark due to its high intensity, minimal overlap, and reliable response across the evaluated concentrations.^4^ Table 2 summarizes the AEF obtained for each vibrational mode, using 1 × 10^–4^ M MB as the *C*_ref_ and comparing it to SERS signals at 1 × 10^–8^ M.

**Table 2:** Analytical Enhancement Factor (AEF), Standard Deviation (SD), and Relative Standard Deviation (RSD) Calculated for the 1618 cm^–1^ Raman Mode of the MB at 1 × 10^–4^to 1 × 10^–8^ Using 1 × 10^–4^ M as the Reference

methylene blue concentration (M)	1618 (cm^–1^) Raman mode			
	intensity (a.u.)	SD (a.u.)	RSD	analytical enhancement factor (AEF)
1 × 10^–4^ (*C*_ref_)	29 (*I*_ref_)	4		
1 × 10^–4^ (SERS)	20,810	1332	6%	7 × 10**^2^**
1 × 10^–6^ (SERS)	8961	627	7%	3 × 10^5^
1 × 10^–8^ (SERS)	3727	309	8%	1 × 10**^6^**

Notably, the enhancement factor for the 1618 cm^–1^ band reached 1 × 10^6^, a competitive value within the range reported for AgNP-decorated TiO_2_ nanotube substrates fabricated by solution-based methods.^4,64,65^ Although higher AEFs (>10^7^) and lower detection limits (down to 10^–12^ M) have been reported for more complex or vacuum-based architectures, the magnitude obtained here underscores the efficiency and simplicity of the electrodeposition protocol. It highlights the BTNTs/AgNP platform as a robust and scalable SERS substrate.

To assess signal uniformity, SERS spectra were collected from randomly selected points across the same optimized substrate. The spectra exhibited low variation in peak intensity, indicating good intrasample uniformity. This was further supported by the calculation of relative standard deviation (RSD)^66^ values for the main Raman band (1618 cm^–1^). As shown in Table 2, the RSD value remained below 10% confirming the homogeneous distribution of plasmonic hotspots over the substrate surface. Moreover, the intersample reproducibility was also demonstrated, with standard deviation and RSD values of 357 au and 9% (from three independent replicates), respectively, for the same Raman mode.^67,68^

To further assess the analytical performance of the system, the limit of detection (LoD) was estimated from a calibration curve constructed using the 1618 cm^–1^ peak intensities of MB at four concentrations. The data, plotted in semi-log scale (intensity vs log­(concentration), Figure 8), followed a linear trend. Linear regression in this regime yielded the calibration (eq 8):

$$I_{\mathrm{LoD}}=3471\times \log_{10}\left(C\right)+31296$$ 8

where *I*_LoD_ is the Raman intensity (a.u.) and *C* is the analyte concentration (M). Using the criterion *I*_LoD_ = *I*_blank_ + 3 *SD*_blank_,^66^ where the blank spectrum corresponds to MB measured on the substrate without AgNPs, thus representing the baseline response without SERS enhancement. Here, 3471 and 31,296 represent the slope and intercept of the calibration curve, respectively. Based on this analysis, the LoD was 1 × 10^–9^ M.

**Figure 8:**
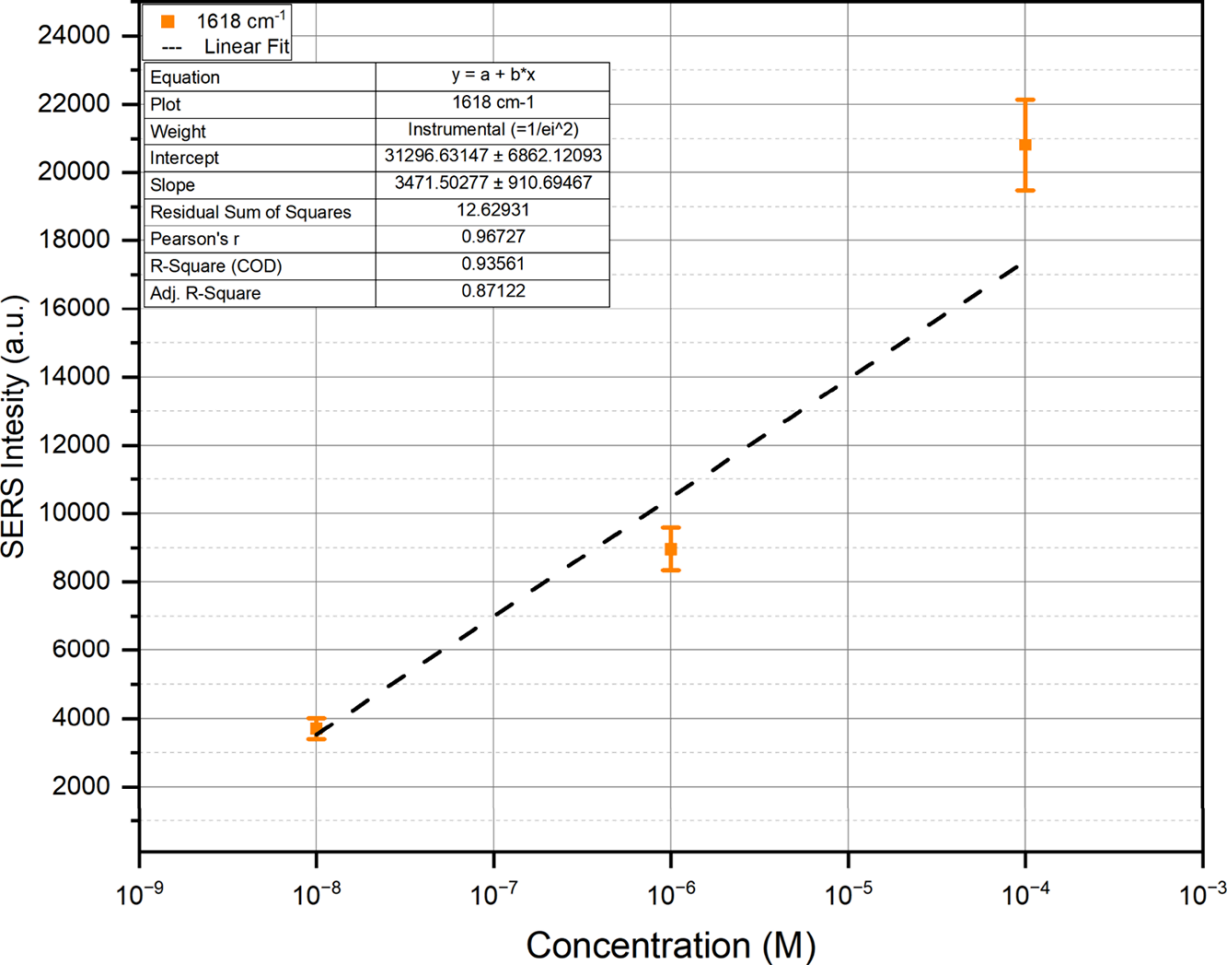
Calibration curve for the 1618 cm^–1^ band (semi-log scale). The LoD was estimated at 1 × 10^–9^ M.

Table 3 compares Ag-decorated TiO_2_ substrates in terms of architecture, fabrication route, analyte, AEF, and limit of detection (LoD). Under our optimized pulsed-current deposition, the BTNTs/AgNPs platform achieves an AEF of 1 × 10^6^ using 1 × 10^–8^ M concentration for methylene blue. Although some vacuum-based or chemically intensive routes report lower LoD, our method offers a cost-effective and scalable alternative that avoids complex tooling while delivering competitive performance suitable for practical monitoring scenarios.

**Table 3:** Comparative Performance of Ag-Decorated TiO_2_ Nanostructured SERS Substrates^a^

substrate composition	synthesis method	analyte	AEF	LoD (M)	ref
TiO_2_ nanotubes and nanograss/AgNPs	constant voltage anodization/DC magnetron sputtering	MB	1 × 10^5^	1 × 10^–12^	^65^
TiO_2_ nanotubes/AgNDs	constant voltage anodization/constant current electrodeposition	MB		1 × 10^–5^	^69^
TiO_2_ nanotubes/AgNPs	constant voltage anodization/thermal evaporation	MB	1 × 10^5^	1 × 10^–8^	^70^
TiO_2_ nanosheet/AgNPs	hydrothermal method/DC magnetron sputtering	R6G	1 × 10^5^	8 × 10^–9^	^71^
TiO_2_ nanorods/AgNPs	chemistry method	MG	4 × 10^5^	1 × 10^–12^	^72^
BTNTs/AgNPs	alternating voltage anodization/pulse current electrodeposition	MB	1 × 10^6^	1 × 10^–9^	this work
TiO_2_ nanotubes/AgNPs	arc ion plating method/DC magnetron sputtering	R6G	1 × 10^9^	1 × 10^–8^	^73^
TiO_2_ nanospheres/AgNPs	commercial nanospheres/ultraviolet light-induced	R6G	7 × 10^10^	1 × 10^–12^	^74^

a R6G: Rhodamine 6G; MB: methylene blue; and MG: malachite green.

The fabrication route presented here—potentiostatic anodization under alternating voltage (60/20 V) combined with pulsed-current Ag electrodeposition—offers intrinsic scalability, as both processes are low-cost, solution-based, and adaptable to large-area substrates without the need for vacuum systems. This contrasts with sputtering and evaporation techniques, where high infrastructure cost and limited throughput restrict practical deployment.

Furthermore, the 785 nm excitation wavelength was deliberately selected because it minimizes fluorescence background and prevents photoinduced reactions of methylene blue (MB), which occur at shorter excitation wavelengths. As MB has a strong absorption band between 600–700 nm, excitation at 532 or 633 nm can trigger reduction to leucomethylene blue and demethylation, leading to spectral variability and unreliable detection. In contrast, 785 nm lies outside this absorption range, providing stable Raman spectra and avoiding photochemical degradation. This interpretation is supported by the recent study of Kopal et al.,^75^ who systematically compared MB SERS responses using 532, 633, 785, and 1064 nm excitations. They demonstrated that while shorter wavelengths enhance chemical contributions and photoreactions, the 785 nm laser produces reproducible spectra dominated by electromagnetic enhancement, making it the most reliable choice for analytical applications. Moreover, 785 nm excitation is widely employed in the SERS literature for MB, which allows direct comparison of our results with previous reports.

## 4 Conclusions

In summary, AgNP-decorated BTNTs fabricated through alternating-voltage anodization and pulsed-current electrodeposition demonstrated reliable experimental SERS detection of methylene blue down to 1 × 10^–8^ M with an AEF of 1 × 10^6^, and a theoretical LoD 1 × 10^–9^ M. Unlike conventional continuous-current deposition, which often leads to uncontrolled particle agglomeration and reduced hotspot density, the pulsed regime employed here suppresses overgrowth while exploiting the segmented BTNTs morphology to promote uniform nucleation and efficient hotspot distribution. This integration between structural segmentation and nanoparticle polydispersity provides a reproducible and scalable route toward cost-effective SERS platforms. Beyond methylene blue, the method holds promise for detecting a diverse range of analytes relevant to environmental monitoring and biomedical diagnostics, highlighting its potential as a practical and scalable alternative to complex and high-cost fabrication strategies.

## Supplementary Material



## Data Availability

The original contributions presented in this study are included in the article. Further inquiries can be directed to the corresponding authors.

## Notes

Additional SEM images illustrating the morphological evolution of Ag deposits obtained under different pulsed-current densities; comparative analysis between bamboo-like TiO_2_ nanotubes and smooth-walled TiO_2_ nanotubes is also provided to highlight structural differences (PDF)
