# Supplementary material for: Pulsed Current Electrodeposition of Ag Nanoparticles on Bamboo-like TiO2 Nanotubes for Surface-Enhanced Raman Scattering Substrates
Source: ACS Omega. 2025 Dec 11;10(50):62257–67. doi: 10.1021/acsomega.5c10021 (PMC12750385; doi:10.1021/acsomega.5c10021)
Supplement: Supplementary file 1 [file ao5c10021_si_001.pdf]

# Supplementary Material

# Pulsed Current Electrodeposition of Ag Nanoparticles on Bamboo-like $TiO_2$ Nanotubes for SERS Substrates

Marcos Luna Cervantes1*, Ismael Garcia-Ramírez1, Erick Octavio Santos Santiago1; Diana Jiménez Girón1; José Luis Zamora Navarro1; Antonio García Chavez2; Leandro García-González1; Adriana Báez-Rodríguez1; Julián Hernández Torres1, Luis Zamora-Peredo1*

[1]Centro de Investigación en Micro y Nanotecnología, Universidad Veracruzana, Av. Adolfo Ruiz Cortines 455, col. Costa Verde, 94294, Boca del Río, México.

[2]Doctorado en Ciencias e Ingeniería, Universidad Autónoma de Baja California, Carretera Transpeninsular Ensenada - Tijuana 3917, col. Playitas, 22860, Ensenada, México

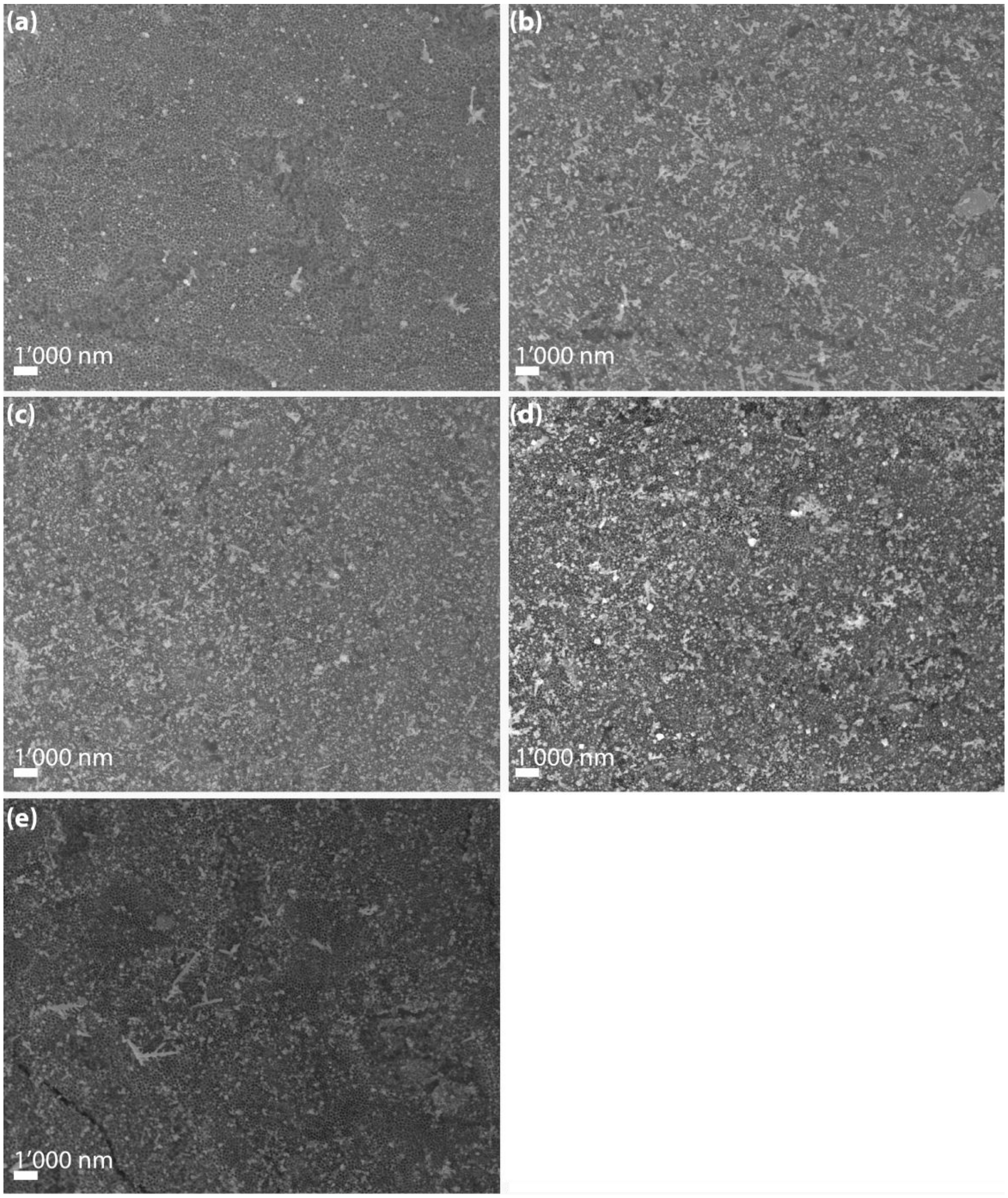


Figure S1. Top-view SEM images showing the morphological evolution of Ag deposits under pulsed-current electrodeposition at 5 mA/cm². (a) 100 cycles; (b) 200 cycles; (c) 300 cycles; (d) 400 cycles; (e) 500 cycles.

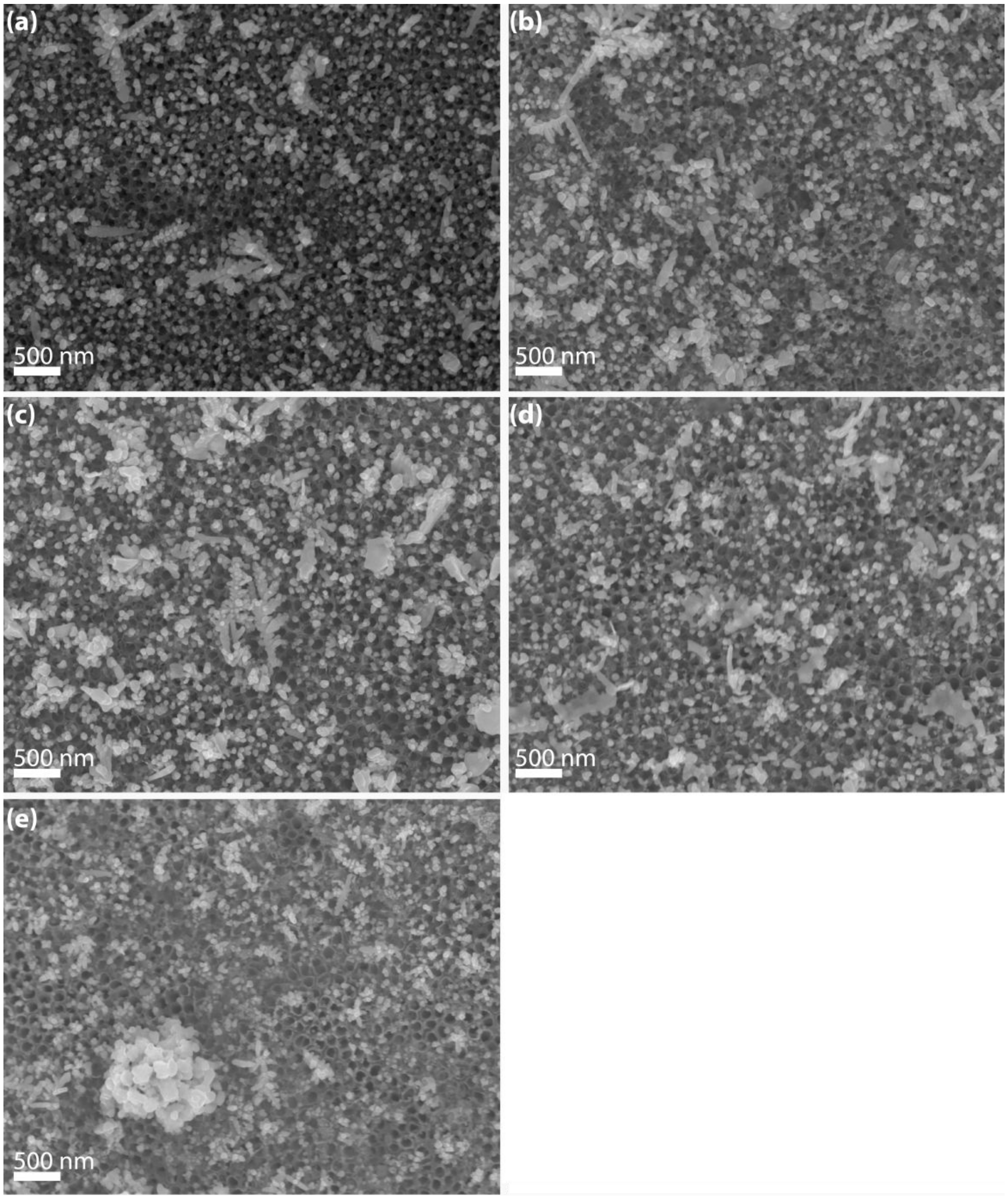


Figure S2. Top-view SEM images showing the morphological evolution of Ag deposits under pulsed-current electrodeposition at (a) 3, (b) 4, (c), 5, (d) 6 , (e) 7  mA/cm² for 100 cycles.

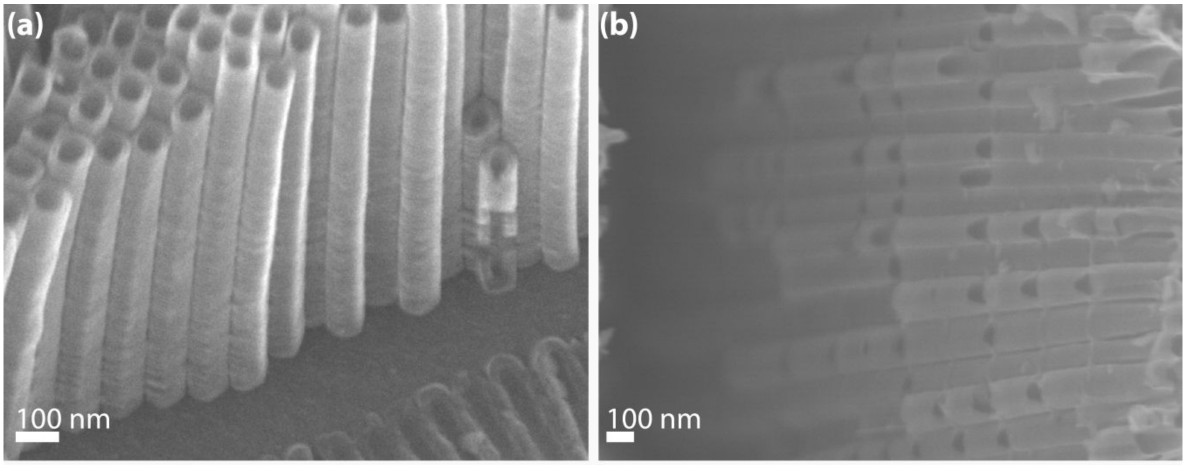


Figure S3. Cross-sectional SEM images comparing the wall morphology of $TiO_2$ nanotube arrays obtained under different anodization regimes. (a) Smooth-walled nanotubes formed under continuous 60 V anodization, showing uniform tube walls with straight, unmodulated profiles. (b) Bamboo-like nanotubes produced under alternating-voltage anodization, exhibiting periodic constrictions along the tube walls characteristic of this morphology.

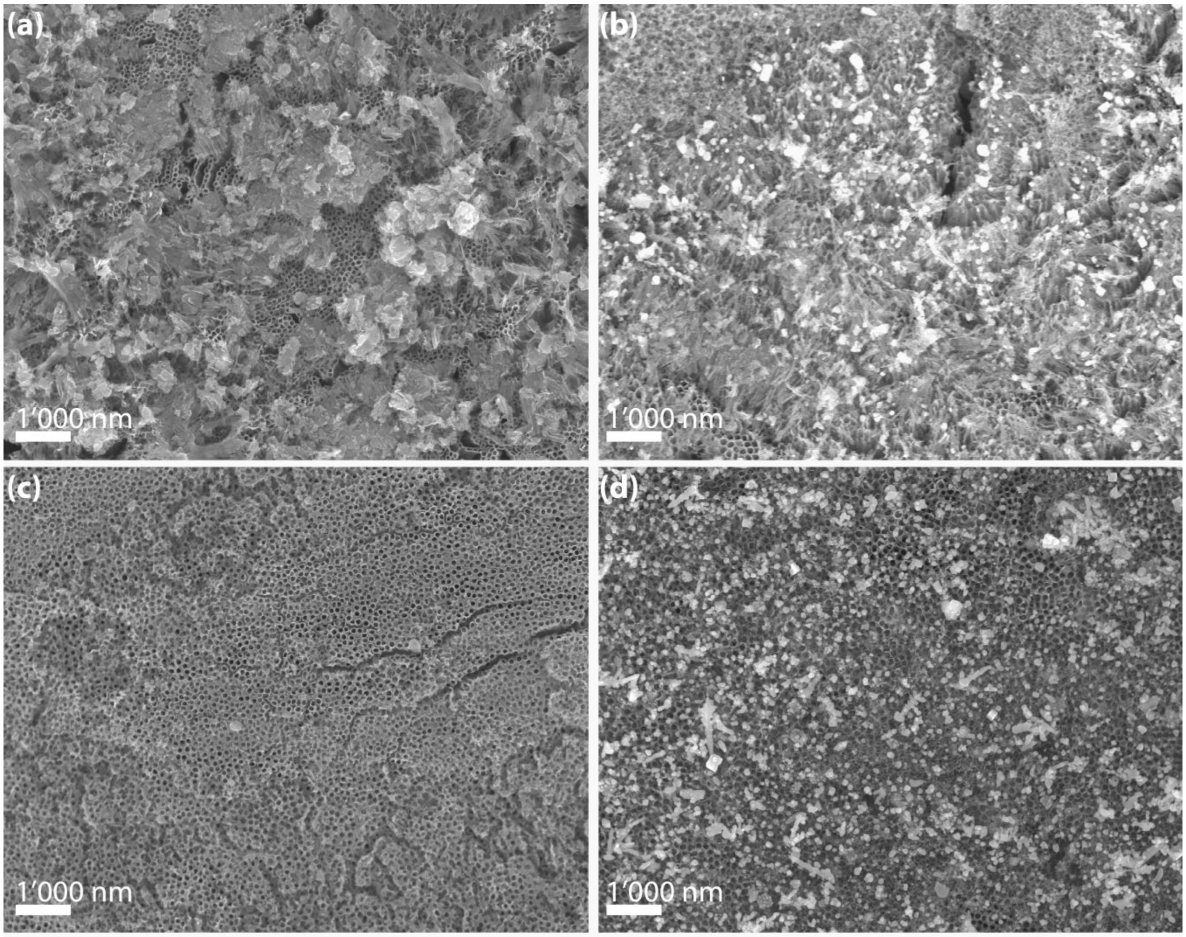


Figure S4. Top-view SEM images of $TiO_2$ nanotube arrays before and after Ag electrodeposition. (a–b) Smooth-walled. (c–d) Bamboo-like nanotubes produced under alternating-voltage anodization.
